# Transfusional iron overload and intravenous iron infusions modify the mouse gut microbiota similarly to dietary iron

**DOI:** 10.1038/s41522-019-0097-2

**Published:** 2019-09-24

**Authors:** Francesca La Carpia, Boguslaw S. Wojczyk, Medini K. Annavajhala, Abdelhadi Rebbaa, Rachel Culp-Hill, Angelo D’Alessandro, Daniel E. Freedberg, Anne-Catrin Uhlemann, Eldad A. Hod

**Affiliations:** 1 Department of Pathology and Cell biology, Columbia University Irving Medical Center, New York, NY USA; 2 Department of Medicine, Columbia University, Irving Medical Center-New York Presbyterian Hospital, New York, NY USA; 3 Columbia Medicine Microbiome and Pathogen Genomic core, Columbia University Irving Medical Center, New York, NY USA; 4 Department of Biochemistry and Molecular Genetics, University of Colorado Denver-Anschutz Medical Campus, Aurora, Colorado USA

**Keywords:** Microbiome, Symbiosis

## Abstract

Iron is essential for both microorganisms and their hosts. Although effects of dietary iron on gut microbiota have been described, the effect of systemic iron administration has yet to be explored. Here, we show that dietary iron, intravenous iron administration, and chronic transfusion in mice increase the availability of iron in the gut. These iron interventions have consistent and reproducible effects on the murine gut microbiota; specifically, relative abundance of the *Parabacteroides* and *Lactobacillus* genera negatively correlate with increased iron stores, whereas members of the Clostridia class positively correlate with iron stores regardless of the route of iron administration. Iron levels also affected microbial metabolites, in general, and indoles, in particular, circulating in host plasma and in stool pellets. Taken together, these results suggest that by shifting the balance of the microbiota, clinical interventions that affect iron status have the potential to alter biologically relevant microbial metabolites in the host.

## Introduction

Iron is a critically important nutrient, required for the viability of virtually all organisms.^1^ Iron deficiency is the most common form of malnutrition worldwide, increasing the risks of disability and death in >2 billion people.^2^ Efforts to prevent and correct iron deficiency by iron supplementation, particularly in children in low-income countries with increased exposure to pathogenic bacteria and parasites, have resulted in increased incidence of infection and death.^3–6^ One leading hypothesis to explain how iron supplementation adversely affects outcomes is through modification of the microbiota, promoting the growth of pathogenic bacteria, and hampering the survival of protective microbes.^7^ Indeed, dietary iron fortification of infants in low-income countries does alter the gut microbiota, favoring enteric pathogens, increasing intestinal inflammation and diarrhea, and reducing the efficacy of antibiotic treatment against potential enteropathogens.^8–10^ However, it is not known whether changing systemic iron levels by blood transfusion has similar effects.

Red blood cell transfusion is a prominent, but often inadvertent method of iron delivery. In patients undergoing hematopoietic stem cell transplantations (HSCT), transfusion-induced iron overload is a risk factor for poorer outcomes.^11–13^ In particular, transfusional iron overload is associated with an increased incidence of infection in the transplant setting.^14–16^ Furthermore, owing to patient blood management efforts to reduce adverse effects of red blood cell transfusions, intravenous iron is increasingly administered to anemic patients with iron deficiency.^17^ Nonetheless, it is unknown whether these interventions influence the gut microbiota similarly to dietary iron manipulations.

In this study, we generated a mouse model of transfusional iron overload to investigate the impact of transfusions on iron-associated alterations to commensal flora in the gut. Furthermore, we compared these results with those obtained in murine models of iron manipulations using dietary and intravenous approaches. Finally, given the iron-mediated changes observed in the gut microbiota, we examined the potential effects on microbial metabolic pathways and altered microbial metabolites circulating in the host. Taken together, these findings represent an important step toward better understanding the role of iron replacement therapies and transfusional iron overload on the gut microbiota and as a possible cause of dysbiosis leading to altered health outcomes.

## Results

### Intravenous iron and chronic transfusions increase fecal iron in mice fed either iron-deficient or iron-replete diet

**Fig. 1:**
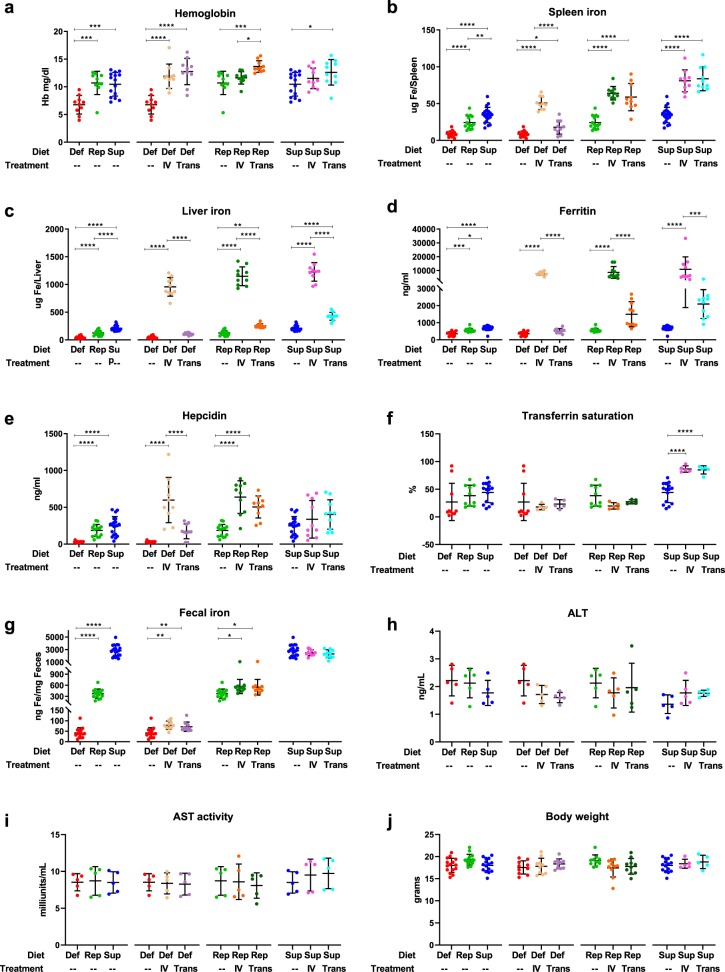
Iron status. **a**–**g** Iron parameters as labeled in each panel, were measured for each mouse on an iron-deficient (Def; *n* = 15), iron-replete (Rep; *n* = 15), or iron-supplemented (Sup; *n* = 20) diet, with intravenous iron infusion (IV; *n* = 10 per group) or weekly blood transfusions (Trans; *n* = 10 per group). Results shown are combined from four separate and complementary experiments. **h**–**i** ALT and AST activity in the liver was measured in the same cohorts of mice for one representative replicate (*n* = 5 per group); **j** Body weight was recorded in three of the complementary experiments. Dot plots graphs report mean ± SD. *p < 0.05, **p < 0.01, ***p < 0.001, ****p < 0.0001 by one-way ANOVA with Tukey’s multiple comparison test

Mice fed an iron-deficient diet became anemic, which was corrected by either intravenous iron or transfusion (Fig. 1a). In mice-fed iron-replete or iron-supplemented diets, chronic transfusion resulted in mild polycythemia. Splenic and hepatic iron content increased with increasing dietary iron, and with intravenous iron or chronic transfusion; however, liver iron increased more with intravenous iron as compared with transfusion (Fig. 1b, c). Serum ferritin levels paralleled liver iron levels (Fig. 1d). Chronic transfusion and intravenous iron increased hepcidin levels in mice fed iron-deficient or iron-replete diets, but did not significantly affect hepcidin levels in mice fed an iron-supplemented diet (Fig. 1e). Mean transferrin saturation was also below 50% for all groups except those on an iron-supplemented diet transfused or infused with iron (Fig. 1f); suggesting that significant levels of labile plasma iron were not produced in these former groups.^18^ The iron content in the diet was reflected in the fecal iron concentration. Furthermore, in parallel with hepcidin levels, chronic transfusion or intravenous iron increased fecal iron concentrations in mice fed iron-deficient or iron-replete diets, but not in mice fed an iron-supplemented diet (Fig. 1g). Furthermore, the various treatments did not induce liver toxicity as evidenced by no difference in alanine and aspartate aminotransferase (ALT and AST, respectively) levels (Fig. 1h, i), body weight (Fig. 1j), and microscopic histologic findings in the liver (Supplementary Fig. 1). Finally, the treatments only had a minor effect on expression levels of pro- and anti-inflammatory molecules in the liver and duodenum (Supplementary Fig. 2).

### Dietary and systemic iron administration affect the microbiota composition

**Fig. 2:**
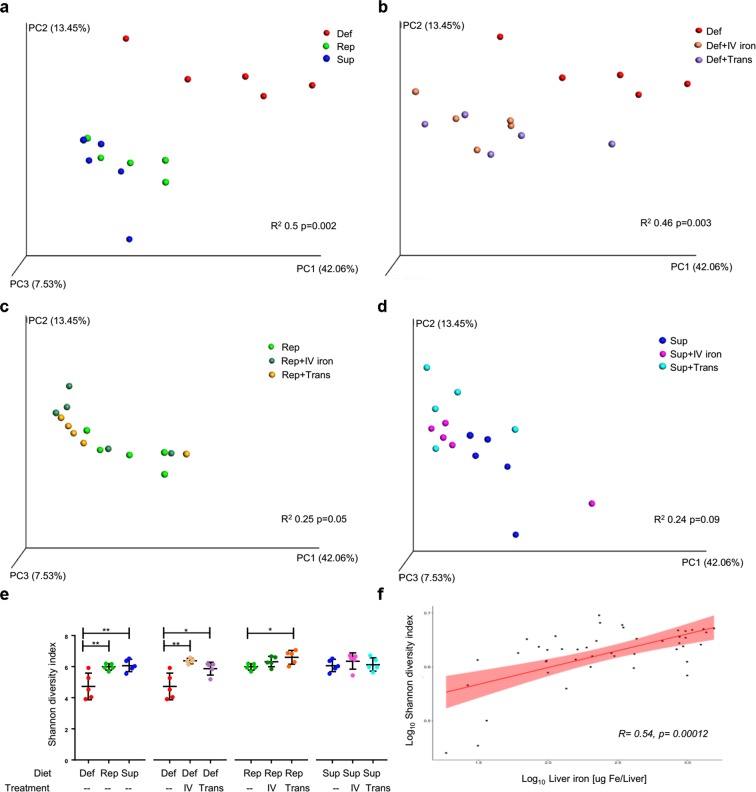
Iron status modulates microbiota diversity. Weighted UniFrac Principal Coordinate Analysis (PCoA) plot of a representative experiment of cohorts of mice **a** fed an iron-deficient (Def), iron-replete (Rep) or iron-supplemented (Sup) diet; **b** fed an iron-deficient diet and either infused with intravenous iron (Def+IV iron) or chronically transfused (Def+Trans); **c** fed an iron-replete diet and either infused with intravenous iron (Rep+IV iron) or chronically transfused (Rep+Trans); and **d** fed an iron-supplemented diet and either infused with intravenous iron (Sup+IV iron) or chronically transfused (Sup+Trans). **e** Shannon alpha diversity index of the mice represented in **a**–**d** and **f** Spearman correlation dot plot comparing logarithm of Shannon alpha diversity index and liver iron for each mouse in a representative experiment; *n* = 5 mice per group; *p < 0.05, **p < 0.01

The dietary iron content affected microbial alpha- and beta diversity, as measured by Shannon index and weighted UniFrac, respectively (Fig. 2a–e, Supplementary Table 1). When stratifying by dietary iron content, systemic iron administration by intravenous infusion or chronic transfusion affected both alpha- and beta diversity; however, this effect became less significant as the dietary iron content increased. Furthermore, alpha diversity was positively correlated with liver iron levels regardless of the diet and how iron was delivered systemically (Fig. 2f). Finally, 16 S copy number analysis of cecal content revealed only minor differences in bacterial load among the groups (Supplementary Fig. 3). Taken together, these results suggest that systemic iron administration, by either transfusion or intravenous iron, modifies the murine gut flora.

### Iron levels negatively correlate with bacteria belonging to the *Lactobacillus* and *Parabacteroides* genera and positively correlate with bacteria belonging to the Clostridia class

**Fig. 3:**
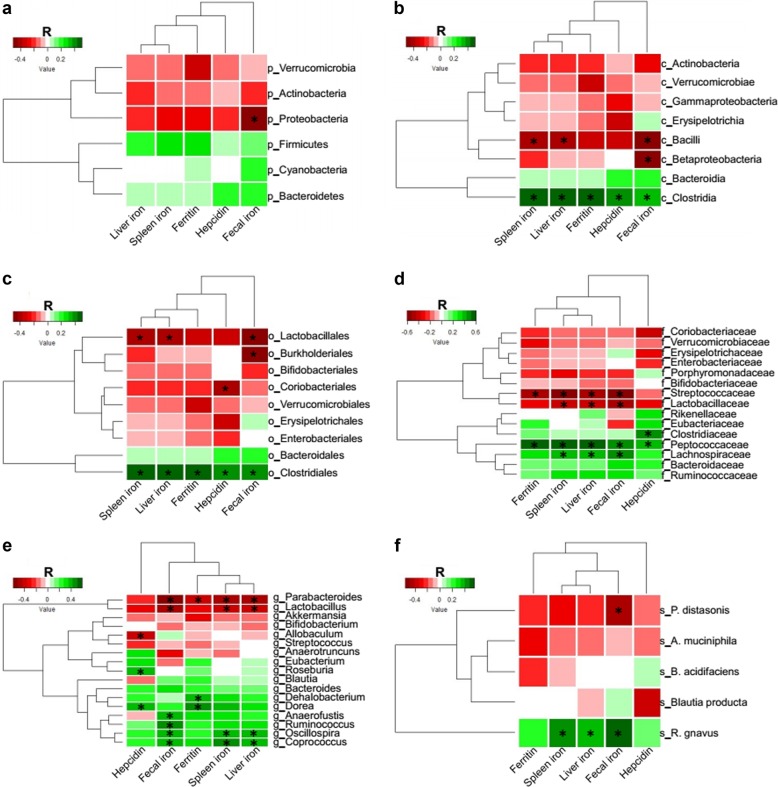
Correlation of bacterial taxa with iron status. Heatmap of Spearman’s correlation between iron parameters and bacterial taxonomic levels of all mice combined from four experiments. Red indicates a negative and green a positive correlation (color intensity reflective of Spearman’s r). **a** p_ = phylum, **b** c_ = class, **c** o_ = order, **d** f_ = family, **e** g_ = genus, **f** s_ = species. *p < 0.0001 for the Spearman’s correlation

To investigate whether iron levels per se affect specific bacteria, we performed a Spearman’s correlation analysis between iron measures (e.g., fecal, liver and spleen iron, serum ferritin and hepcidin) and the relative abundance of bacteria OTUs aggregated at different taxonomic rank. Only bacterial taxa detected in all four complementary experiments were included in this analysis (Supplementary Data 1). *Lactobacillus* and *Parabacteroides* genera exhibited the strongest negative association with iron status (Fig. 3, Supplementary Data 2). Conversely, only bacteria belonging to the Clostridia class positively correlated with iron status.

### Iron status modifies the gut microbiota independently of the administration route

**Fig. 4:**
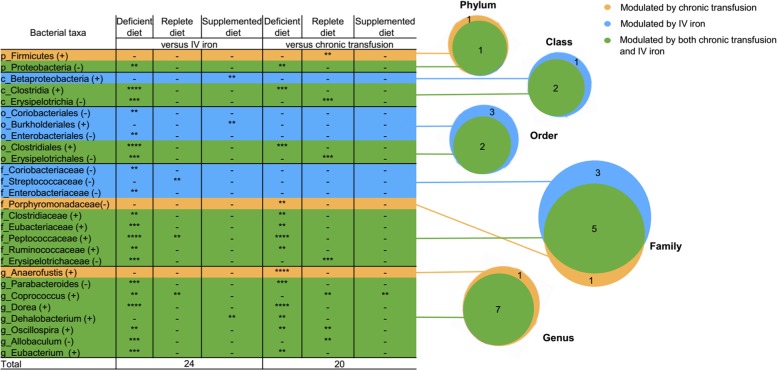
LEfSe analysis of bacterial taxa modulated by either intravenous iron infusion or chronic transfusion. Table and corresponding Venn diagrams of the total number of bacteria at different taxonomic rank increased (+) or decreased (−) by intravenous iron infusion (IV Iron; blue), chronic transfusions (yellow), or both (green) in the three different diet groups, as labeled. p_ = phylum, c_ = class, o_ = order, f_ = family, g_ = genus; *p < 0.05, **p < 0.01, ***p < 0.001, ****p < 0.0001 of Least Discriminant Analysis effect size (LDA) score

To investigate whether administration of iron by intravenous infusion or chronic transfusion differentially affects the microbiota, we performed Linear Discriminant Analysis (LDA) effect size (LEfSe)^19^ on the same bacterial data set used for the Spearman’s correlation analysis. The gut microbiota composition of cohorts of mice receiving intravenous iron or chronic transfusion were compared with cohorts of untreated mice for each respective diet. Differentially abundant bacterial taxa at different taxonomic rank with a *p* value of 0.01 and LDA score higher than 2.0 were identified. Twenty-four and 20 bacteria at different taxonomic rank were modified by intravenous iron infusion and chronic transfusion, respectively (Fig. 4). Among these, 17 were common to both iron treatments.

**Fig. 5:**
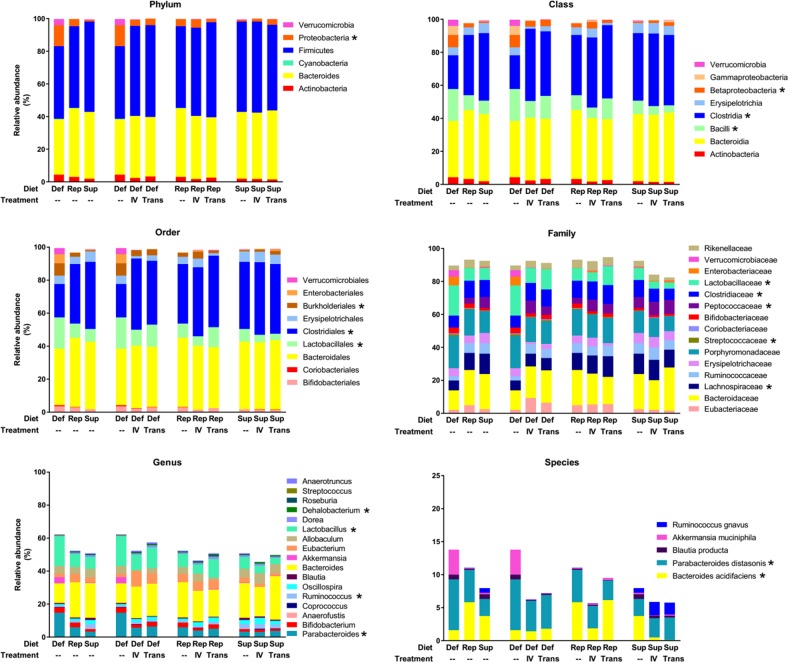
Relative abundance of all taxonomic ranks. Relative abundance of all taxa from four combined experiments in cohorts of mice fed an iron-deficient (Def), iron-replete (Rep) or iron-supplemented diet (Sup), and from cohorts of mice infused with intravenous iron (IV) or chronically transfused (Trans). Each bar represents the mean of the relative abundance of the corresponding taxa, across four complementary experiments (*n* = 10–20 mice per group); * denotes bacteria modulated by diet and/or treatments (IV iron or chronic transfusion) with *p* < 0.0001 from mixed model analysis

To confirm the LEfSe results and to account for the variability between experiments, a mixed model analysis was performed, considering diet and parenteral treatments as fixed effects and each experiment as a random effect. Using mice fed an iron-replete diet as the control, 18 bacterial taxa emerged as being modified by diet and/or parenteral iron treatment with a *p* value of <0.0001 (Fig. 5, Table 1). Among these, 17 bacterial taxa were affected by intravenous iron administration or chronic transfusion in a similar fashion to the dietary iron supplementation. Taken together, these results suggest that the delivered iron plays the dominant role in modifying the microbiota, irrespective of the route of administration.

**Table 1:** Mixed model analysis of relative abundance of bacterial taxa

		Fixed effects			
Taxonomic rank	Denomination	Diet		Treatment	
		Def	Sup	IV iron	Transfusion
Phylum	Proteobacteria	3.91**	−2.25	−2.41*	−2.98*
Class	Bacilli	5.97***	−4.54*	−6.98***	−3.84*
	Clostridia	−8.16***	2.54	10.73****	9.19****
	Betaproteobacteria	2.63***	−0.9	−0.36	−0.88
Order	Lactobacillales	6.02***	−4.65*	−7.24****	−4.18*
	Clostridiales	−7.72***	2.97	10.46****	9.03****
	Burkholderiales	2.63***	−0.9	−0.36	−0.88
Family	Lachnospiraceae	−4.12****	0.67	0.51	0.22
	Streptococcaceae	0.26**	−0.23*	−0.36****	−0.19*
	Peptococcaceae	−1.41	2.45**	3.45****	2.25**
	Clostridiaceae	−1.77**	2.39**	2.81****	2.44***
	Lactobacillaceae	5.76***	−4.42*	−6.89***	−3.99*
Genus	Parabacteroides	4.56****	−1.80*	−3.76****	−2.96**
	Ruminococcus	−0.14	1.32****	0.14	0.04
	Lactobacillus	5.76***	−4.42*	−6.89***	−3.99*
	Dehalobacterium	−0.04	0.12	0.30***	0.34****
Species	Bacteroides acidifaciens	−3.17****	−1.18	0.04	1.46*
	Parabacteroides distasonis	2.14**	−1.07	−1.29*	−1.14

Parameter estimates of taxa modulated by dietary iron, intravenous (IV) iron, or chronic transfusion with a *p* value < 0.0001 for the test of fixed effects. Positive and negative values indicate increasing and decreasing trend, respectively, when compared with cohorts of mice fed an iron-replete diet alone. *p < 0.05, **p < 0.01, ***p < 0.001, ****p < 0.0001

**Fig. 6:**
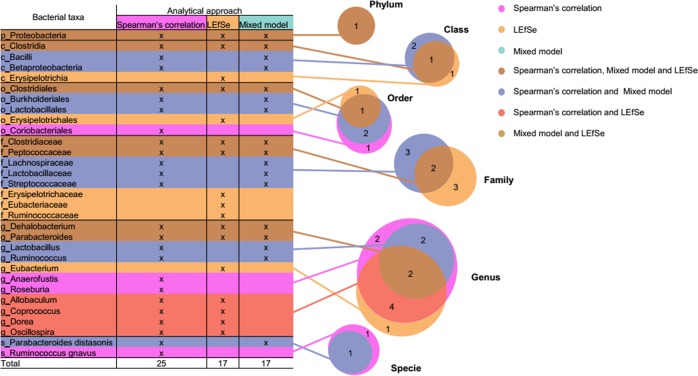
Combination of all three approaches used to analyze the effects of iron on the microbiome. Table and Venn diagram indicating the distribution of the bacteria at different taxonomic rank modified by intravenous iron and chronic transfusion that were identified by Spearman’s correlation (25), LEfSe (17) and mixed model analysis (17) from the four complementary experiments. Taxa highlighted in dark gold were identified by all three approaches. p_ = phylum, c_ = class, o_ = order, f_ = family, g_ = genus, s_ = species

Combining the three different approaches to identify bacterial taxa modified by iron status (i.e., Spearman’s correlation, Fig. 3; LEfSe analysis, Fig. 4; mixed effects model, Fig. 5, Table 1), Proteobacteria phylum, Clostridia class in phylum Firmicutes, and its dependent bacterial taxa Clostridiales order, *Clostridiaceae* and *Peptococcaceae* families and *Dehalobacterium* genus, and *Parabacteroides* genus in phylum Bacteroidetes, were most consistently associated with iron levels and iron treatment (Fig. 6).

### Iron status affects the predicted functional profile of the gut microbiota

To predict whether iron levels affect the functional profile of the gut microbiota, PICRUSt analysis was performed to predict potential pathways affected by iron availability in the gut. Based on fecal iron content, we compared the cohort of mice fed an iron-deficient diet with cohorts fed an iron-replete diet, or an iron-deficient diet with intravenous iron administration, or an iron-deficient diet with chronic transfusion. At Kyoto Encyclopedia of Genes and Genomes (KEGG) level 3, and as compared with mice fed an iron-deficient diet, 100 pathways were predicted to be significantly different in mice fed an iron-replete diet. Furthermore, 101 and 54 pathways were predicted in the cohorts of mice infused with intravenous iron or chronically transfused, respectively. Among these, 51 pathways were shared by all three cohorts of mice with increased iron level as compared with the iron-deficient mice (Supplementary Fig. 4a, b). PCA analysis confirmed the overlap and similarity of the predicted pathways at KEGG level 3 and demonstrated that the mice in the cohort receiving only the iron-deficient diet cluster separately from the three groups with increased fecal iron (Supplementary Fig. 4c). Thus, the PICRUSt analysis predicted that iron status may functionally affect microbial metabolism and prompted an exploration regarding whether microbial metabolites are modified by iron status.

### Untargeted metabolomics analysis reveals that metabolites of tryptophan degradation are negatively associated with increasing iron availability

**Fig. 7:**
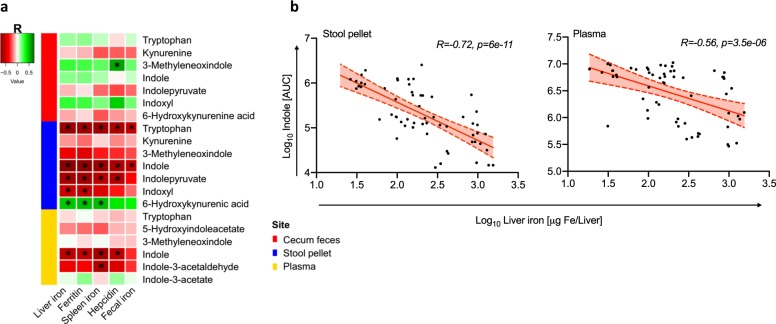
Iron modulation of “Indole and Tryptophan” pathway metabolites. **a** Spearman’s correlation of metabolites belonging to the “Indole and Tryptophan” pathway in cecal feces, stool pellets, and plasma as correlated to standard iron parameters. **b** Correlation between Log_10_ of quantified indole Area Under the Curve (AUC) and Log_10_ of liver iron in stool pellets and plasma, as indicated. *p < 0.0001 for the Spearman’s correlation

To investigate metabolic changes associated with iron status, untargeted metabolomics analysis was performed on cecal feces, stool pellets, and plasma. Spearman’s correlation between detected metabolites and iron parameters revealed that increasing iron levels correlate with levels of multiple metabolites (Supplementary Fig. 5, Supplementary Data 3–5). Of the microbially produced metabolites examined, indoles, which are derived from tryptophan degradation with protective effects on the gut mucosa,^20,21^ exhibited the strongest negative correlation with fecal and plasma iron levels (Fig. 7).

## Discussion

Iron is a vital element for biological functions in both eukaryotic and prokaryotic cells. Increased iron availability affects bacterial virulence^22,23^ and changes the balance of commensal bacteria in the gut, promoting growth of more pathogenic *Enterobacteriaceae* at the expense of protective bacteria that are less dependent on iron (e.g., *Lactobacilli*).^7,24,25^ However, the effects of iron delivered systemically, such as by intravenous iron therapy or red blood cell transfusion, have not been fully characterized.^26^ In the current study, although dietary iron had the greatest impact on fecal iron levels, both intravenous iron infusion and chronic transfusion affected fecal iron levels when murine diets were not iron supplemented. Fecal iron was associated with alpha and beta diversity changes in the composition of the murine gut microbiota; furthermore, intravenous iron infusion and chronic red blood cell transfusion had similar effects. Taken together, these findings suggest that the delivery of iron, irrespective of the route of administration, has strong effects on the murine gut microbiota.

Owing to the variability of the baseline microbiota in repeat experiments,^27–29^ we identified bacterial taxa that were similarly modulated by iron across four complementary experiments, analyzing a total of 110 mice, and integrating three different analytical approaches (i.e., LEfSe, mixed model analysis, and Spearman’s correlation); this was done to minimize the effects of non-controllable variables on the microbiota. Using this strategy, bacterial taxa belonging to Clostridia class (Clostridiales order, *Clostridiaceae* and *Peptococcaceae* families, and *Dehalobacterium* genus) were found to proliferate with increasing levels of iron; in contrast, *Parabacteroides* genus in the Bacteriodetes phylum and Proteobacteria phylum decreased in abundance with increasing iron availability. Finally, there was a significant negative correlation between bacteria in the *Lactobacillaceae* family and iron status using two of the three analytical approaches (i.e., Spearman’s correlation and mixed model analysis). *Clostridiaceae*, *Lactobacillaceae*, and *Parabacteroides* were previously shown to be modified by oral iron;^7–9,30,31^ the current results extend these findings to systemic iron administration. In contrast, very little is known about the relationship between iron and members of the *Peptococcaceae* family, Gram-positive obligate anaerobic bacteria, which are positively associated with production of trimethylamine N-oxide (TMAO),^32^ a metabolite that increases thrombotic risk by enhancing platelet activation.^33^ Thus, in this study, we demonstrate that this bacterial taxa has a strong positive association with iron availability.

Although we did not observe major differences in systemic and duodenal inflammation (Supplementary Fig. 2) in these wild-type and healthy mice, the protective role of *Lactobacillaceae* and *Parabacteroides* in the gut has been shown in a mouse model of dextran sulfate sodium-induced colitis, where the presence of *Lactobacillaceae* and *Parabacteroides* have been associated with increased intestinal barrier function and reduced intestinal inflammation, respectively.^34,35^ Furthermore, in both murine and human studies, *Lactobacillus* and *Parabacteroides* are beneficial in the setting of HSCT and acute graft-versus-host disease (GVHD), the latter a dangerous adverse outcome of HSCT.^36–38^ Thus, our findings that *Lactobacillus* and *Parabacteroides* are reduced by chronic transfusion are particularly relevant in the context of HSCT, considering that these patients often become iron overloaded as a result of their transfusion requirements and that this iron overload portends worse outcomes.^11,12^ Finally, patients with chronic kidney disease are often treated with intravenous iron and microbiome studies in these patients suggest dysbiosis with a particular reduction in *Lactobacillus* species.^39,40^ Whether iron is responsible for these findings in patients with chronic kidney disease has yet to be investigated.

The gut microbiota affects the host by producing various metabolites. In this study, we show that these iron-mediated microbiota changes are also associated with changes in metabolites produced by microbiota, particularly indoles, which originate from the microbial metabolism of tryptophan,^41,42^ in both stool pellets and plasma. Using untargeted metabolomics profiling, we demonstrate that increased iron levels are associated with decreased indole metabolites. Indoles protect the gut mucosa, by increasing tight-junction protein expression, reducing epithelial inflammation,^20^ and conferring resistance to dextran sodium sulfate-induced colitis in mice.^21^ Furthermore, acting via Type I interferon, indoles reduce GVHD pathology and mortality in mice^43^ and low levels of urinary 3-indoxyl-sulfate are associated with higher transplant-related mortality and lower overall survival in allogeneic HSCT patients.^44^ Whether these iron-mediated alterations to the commensal microbiota and to microbiota-derived metabolites, such as indoles, induce some of the clinical effects associated with iron overload, remains unknown and requires further investigation.

Although iron metabolism in laboratory mice is generally similar to that of humans,^45^ there are notable differences. For example, mice do not absorb heme iron as well as humans.^46^ Furthermore, mice have greater iron losses relative to iron stores,^45^ and have a more active iron excretion pathway as compared with humans.^47–49^ Thus, a limitation of this study is that the increases in fecal iron content observed with chronic transfusion or intravenous iron infusion in this murine model may not fully translate to humans. However, we hypothesize that increasing iron status in humans may lead to an increased iron concentration in the gut lumen through the action of hepcidin, the iron-regulatory hormone,^1^ which would prevent dietary iron absorption, resulting in increasing iron availability to gut microbiota through sloughing of iron-loaded enterocytes into the gastrointestinal lumen at the end of their lifespan. Furthermore, limited bile excretion pathways may exist in iron-overloaded humans.^47,50,51^ To determine how well these murine findings translate to the human setting, we are currently conducting a human study (ClinicalTrials.gov # NCT02990988) in which stool samples are collected from iron-deficient volunteers before and after repletion by intravenous iron infusion. Finally, another limitation of our study is that other forms of iron administration were not tested; however, chronic transfusion, oral iron supplementation, and intravenous iron represent the main routes of changing iron status in patients.

In conclusion, we observe that intravenous iron infusions and chronic transfusion, in a mouse model, modified fecal iron levels, and the gut microbiota in similar ways to those observed with dietary iron supplementation. These changes affected multiple predicted functional pathways in gut microbiota and, most importantly, metabolite levels, such as indoles, in feces and plasma. Although the human clinical implications of these findings in a murine model are yet to be determined, the fact that indoles are known to affect clinical outcomes and, in our setting, were modulated by iron, strengthens the importance of better understanding iron-mediated host-microbiota interactions.

## Methods

### Mice

Weanling female wild-type C57BL/6 mice were purchased from Charles River Laboratories (Stone Ridge, NY), housed in a pathogen-free facility, and used at 3 weeks of age. Cohorts of mice were fed ad libitum either an iron-deficient (TD.110592, Envigo, 0–5 ppm Fe), iron-replete (TD. 110593, Envigo, 45 ppm Fe), or iron-supplemented (TD.110594, Envigo, 220 ppm Fe) diet for 6 weeks. Two additional cohorts of mice were generated for each of these dietary groups by intravenous infusion of iron dextran (0.33 mg weekly × 6; Henry Schein Animal Health, Dublin, OH; 2 mg iron total over 6 weeks) or transfusing red blood cells (0.33 mL at 60% hematocrit weekly × 6 using fresh blood collected from wild-type C57BL/6 mice on a regular chow diet; mouse equivalent to 10 human units of blood transfused or 2 mg total iron administered). Following 6 weeks on defined diets, with or without additional iron treatment, all cohorts were euthanized and tissues, blood, and feces from rectum and cecum were collected for analysis. Between 10 and 20 mice per group total, in four complementary experiments were analyzed. For all experiments, only mice on the same diet and provided with the same treatment were cohoused (*n* = 5 per cage), so that coprophagic behavior would not influence the results. All animal studies were approved by the Columbia University Institutional Animal Care and Use Committee.

### 16 S sequencing and copy number, microbiota, and PICRUSt analysis

Cecal fecal samples were collected and DNA extracted using QIAamp power fecal DNA kit (Qiagen). 16 S rDNA V4 or V3–V4 variable regions were amplified by PCR and sequenced using the Illumina MiSeq platform. For the first three experiments, 16 S rDNA V4 sequencing was performed at MR DNA (www.mrdnalab.com, Shallowater, TX, USA) and operational taxonomic units (OTUs) were defined by clustering at 97% similarity. Final OTUs were taxonomically classified using BLASTn against a curated database derived from Greengenes, RDPII and NCBI (www.ncbi.nlm.gov, www.greengenes.lbl.gov, http://rdb.cme.msu.edu). For the fourth experiment, 16 S rDNA V3–V4 sequencing was performed at the Microbiota Core facility at the Columbia University Irving Medical Center (CUIMC). QIIME^52^ was used to cluster OTUs at 97% sequence similarity and aligned against the Greengenes database with noise filtering. A minimum cut off of 10,000 counts for a sample to be analyzed was used for all experiments. The relative proportion of multiple OTU sequences within each sample, mapping to the designated taxonomic classification, was used to perform the mixed model analysis and the pairwise linear discriminant analysis effect size (LEfSe),^19^ excluding the bacterial taxa not detected in all four experiments. 16 S rDNA data were also used for metagenome functional prediction with PICRUSt on the online Galaxy platform (http://huttenhower.sph.harvard.edu/galaxy/).^53^ Sequences were mapped to the Greengenes ver 13.5 database and normalized to check for differences in 16 S rDNA copy number between OTUs. STAMP^54^ software was used to analyze KEGG Level 2 and 3 pathway profiles. Bacterial load was analyzed by qPCR of 16 S rDNA copies using RT2 SYBR green ROX qPCR Mastermix (Qiagen) on an AriaMx Real-Time PCR system as described by Staffas et al.^55^

### Metabolomics

Plasma and weighted cecal feces and stool pellets were collected after 6 weeks of dietary manipulation and/or iron treatment. Blood was obtained via cardiac puncture into heparinized syringes and plasma separated after centrifugation at 2500 × *g* for 10 min at 4 °C. All samples were stored at − 80 °C until further processing. Metabolomics analyses of indole metabolites were performed as previously described.^56^ In brief, stable isotope standards tryptophan (^15^N_2_ - NLM-800–0.25) were purchased from Cambridge Isotopes and samples were thawed on ice, then 20 µL (fluid) or 10 mg (tissue) was extracted with 480 µL or 1 mL respectively, of ice cold extraction buffer (5:3:2 MeOH:MeCN:H2O) containing 0.1 μM each of the heavy standards. Extraction was performed by vigorous agitation at 4 °C for 30 min followed by centrifugation at 12,000 rpm, 4 °C for 10 min A 100 μL aliquot of supernatant was transferred to a glass vial, dried under N_2_ flow, and resuspended in an equal volume of water containing 0.1% (v/v) formic acid. Aqueous extracts were analyzed by ultra-high pressure liquid chromatography-mass spectrometry (UHPLC-MS) on a Thermo Vanquish UHPLC (San Jose, CA) coupled to a Thermo Q Exactive mass spectrometer (Bremen, Germany) via positive electrospray ionization. Solvents were water (phase A) and acetonitrile (phase B) supplemented with formic acid (0.1%) and flow rate was 0.25 mL/min. Metabolites were separated using a Kinetex C18 (Phenomenex, Torrance, CA) column (2.1 × 150 mm, 1.7 um) with a 6 min gradient of 0–2 min 2% B; 2–2.5 min increase to 25% B; 2.5–4 min hold at 25% B; 4–4.01 min decrease to 2% B; 4.01–6 min hold at 2% B. The Q Exactive mass spectrometer was operated in full scan mode over the range of 65–950 m/z. Samples were randomized and a quality control sample was injected every 10 runs. Data analysis was performed using Maven (Princeton University) following file conversion by MassMatrix (Case Western Reserve University). Other metabolites were analyzed through the same platform, with methods and workflows extensively described in prior publications.^57^

### Iron-related measurements

All iron-related measurements were obtained at the time of euthanasia. Non-heme iron of spleen, liver, and stool pellets were determined using a wet ashing procedure.^58^ In brief, the wet weight of samples obtained at necropsy was quantified and portions of liver, spleen, or stool pellets, were placed in 2 ml micro-tubes. Following desiccation at 65 °C for 24 h, 1 ml of acid digestion mixture (3 M hydrochloric acid, 10% trichloracetic acid) was added and samples were heated at 65 °C for an additional 24 h. The acidified sample (50 μl) was then incubated for 30 min with 200 μl of chromogen (1.6 M bathophenanthroline, 2 M sodium acetate, 11.5 M thioglycolic acid). Absorbance at 535 nm of samples and iron standards was measured in duplicate and mean values used for calculating total iron. Iron transferrin saturation in plasma was calculated using Iron/TIBC Reagent Set (Pointe Scientific, Canton, MI). Plasma ferritin, plasma hepcidin, and liver ALT levels in liver were measured by ELISA following the manufacturer’s instructions (Kamiya Biomedical, Seattle, WA; Intrinsic LifeSciences, La Jolla, CA; and Biomatik, Wilmington, DE, respectively). AST was measured in liver using the AST activity assay kit (Sigma Aldrich, St. Louis, MO).

### Histology

At necropsy, liver was removed, fixed overnight with 10% neutral-buffered formalin and embedded in paraffin. Sections were stained with hematoxylin and eosin and images captured using a 10 **×** Olympus objective on an Olympus BX51 microscope and an Infinity HD digital camera (B&B Microscopes Ltd.)

### Quantitative reverse transcriptase PCR

Liver and duodenal tissue (20–50 mg) were collected at necropsy in RNA*later* RNA Stabilization Solution (Invitrogen). After 24 h of incubation at +4 °C, tissues were homogenized in RLT buffer for RNA extraction using RNeasy Plus mini Kit (Qiagen) followed by processing with RT^2^ First strand kit (Qiagen) for cDNA synthesis. Quantitative PCR for *Mcp1* (Fw 5′-CCTGGATCGGAACCAAATGAGATCAG-3′; Rev 5′-AGTGCTTGAGGTGGTTGTGGAA-3′), *Lcn2* (Fw 5′-CCACCACGGACTACAACCAG-3′; Rev 5′-AGCTCCTTGGTTCTTCCATACA-3′), *Saa1/2* (Fw 5′-GCGAGCCTACACTGACATG A-3′; Rev 5′-GGCAGTCCAGGAGGTCTGTA-3′) and *Il10* (Fw 5′-CGGGAAGACAATAACTG-3′; Rev 5′-ATTTCCGATAAGGCTTGG-3′) genes was performed using RT2 SYBR® green ROX qPCR Mastermix (Qiagen) on an AriaMx Real-Time PCR system and normalized to the housekeeping gene G*apdh* (Fw 5′-ATGACTCCACTCACGGCAAAT TC -3′; Rev 5′-ACACCAG TAGACTCCACGACATAC-3′).

### Statistical analysis

Significance among means was calculated using one-way ANOVA with the Tukey Multiple Comparison Test or Kruskal–Wallis test with Dunn’s Multiple Comparison Test, as appropriate. A *p* value of <0.05 was considered significant. Statistical analyses were performed using Prism 7 (GraphPad Software, Inc., La Jolla, CA) and means ± standard deviations (SD) are illustrated in the figures. PermANOVA (Permutational Multivariate Analysis of Variance Using Distance Matrices) test of weighted and unweighted UniFrac distance was calculated with R (version 3.4.2) using the vegan package (*adonis* function). For LEfSe analysis, a *p* value of 0.01 for the Kruskal–Wallis test and LDA score higher than 2.0 were used. SAS studio was used for mixed model analysis and Spearman’s correlation. A conservative *p* value of <0.0001 was considered significant in these analyses to limit false discovery given a Bonferroni correction for examining 400 bacterial taxa. The heatmap and dot plot graphs of Spearman’s correlation were generated using ggplot2 and ggpubr packages in R software, respectively. Venn diagrams describing the overlap between the detected bacterial taxa and functional profiles were computed with Biovenn^59^ and manually edited with Inkscape 0.92 (https://inkscape.org/).

### Reporting summary

Further information on research design is available in the Nature Research Reporting Summary linked to this article.

## Supplementary information

Supplementary Data 1

Supplementary Data 2

Supplementary Data 3

Supplementary Data 4

Supplementary Data 5

Reporting Summary Checklist

Supplementary Figures and Table 1

## Data Availability

The authors declare that all relevant data supporting the findings of the study are available in this article and its Supplementary Information file, or from the corresponding author upon request.

## Supplementary information

**Supplementary information** accompanies the paper on the *npj Biofilms and Microbiomes* website (10.1038/s41522-019-0097-2).
