# Supplementary material for: Transfusional iron overload and intravenous iron infusions modify the mouse gut microbiota similarly to dietary iron
Source: NPJ Biofilms Microbiomes. 2019 Sep 24;5:26. doi: 10.1038/s41522-019-0097-2 (PMC6760189; doi:10.1038/s41522-019-0097-2)
Supplement: Supplementary file 7 — Supplementary Figures and Table 1 [file 41522_2019_97_MOESM7_ESM.pdf]

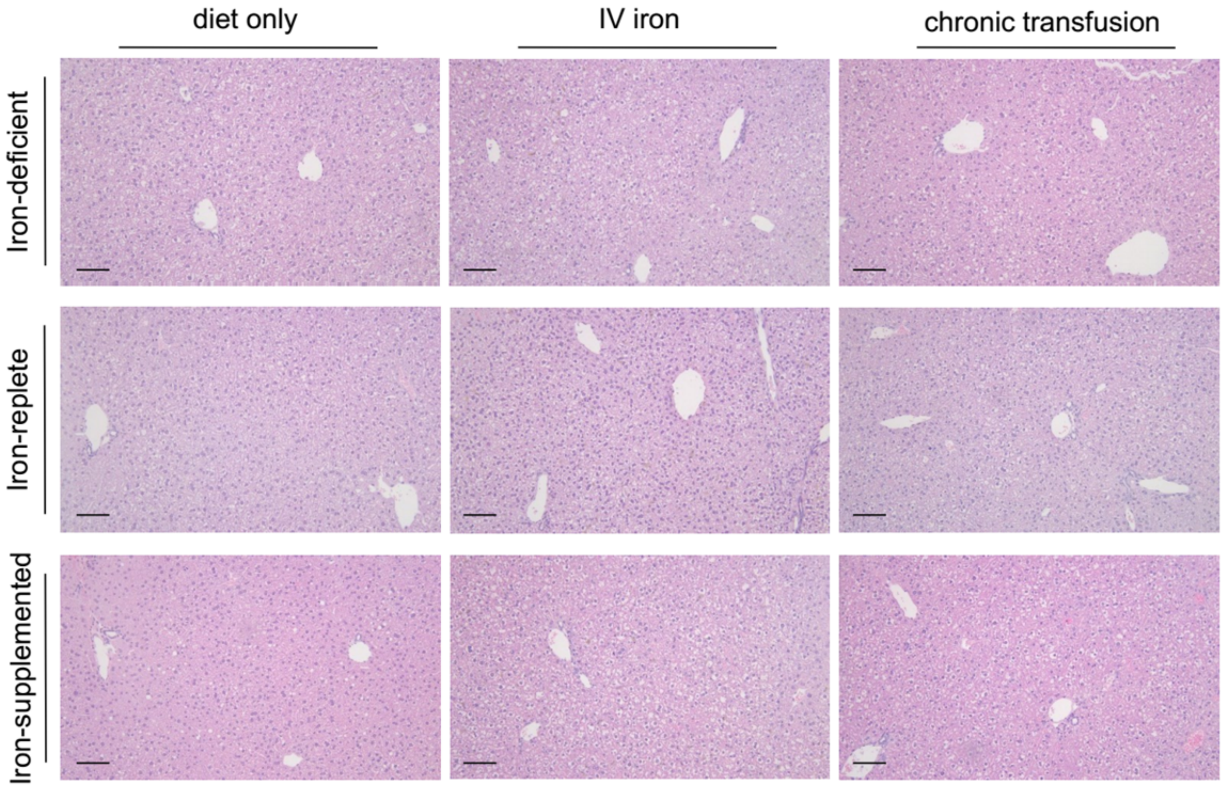

Supplementary Figure 1. Liver histology. Representative images of hematoxylin and eosin (H&E) stained liver histologic sections from groups of mice fed an iron-deficient, replete and supplemented diet. Sections were also examined from cohorts from each diet also infused with intravenous (IV) iron or chronically transfused, as labeled. Scale bars = 50 µm

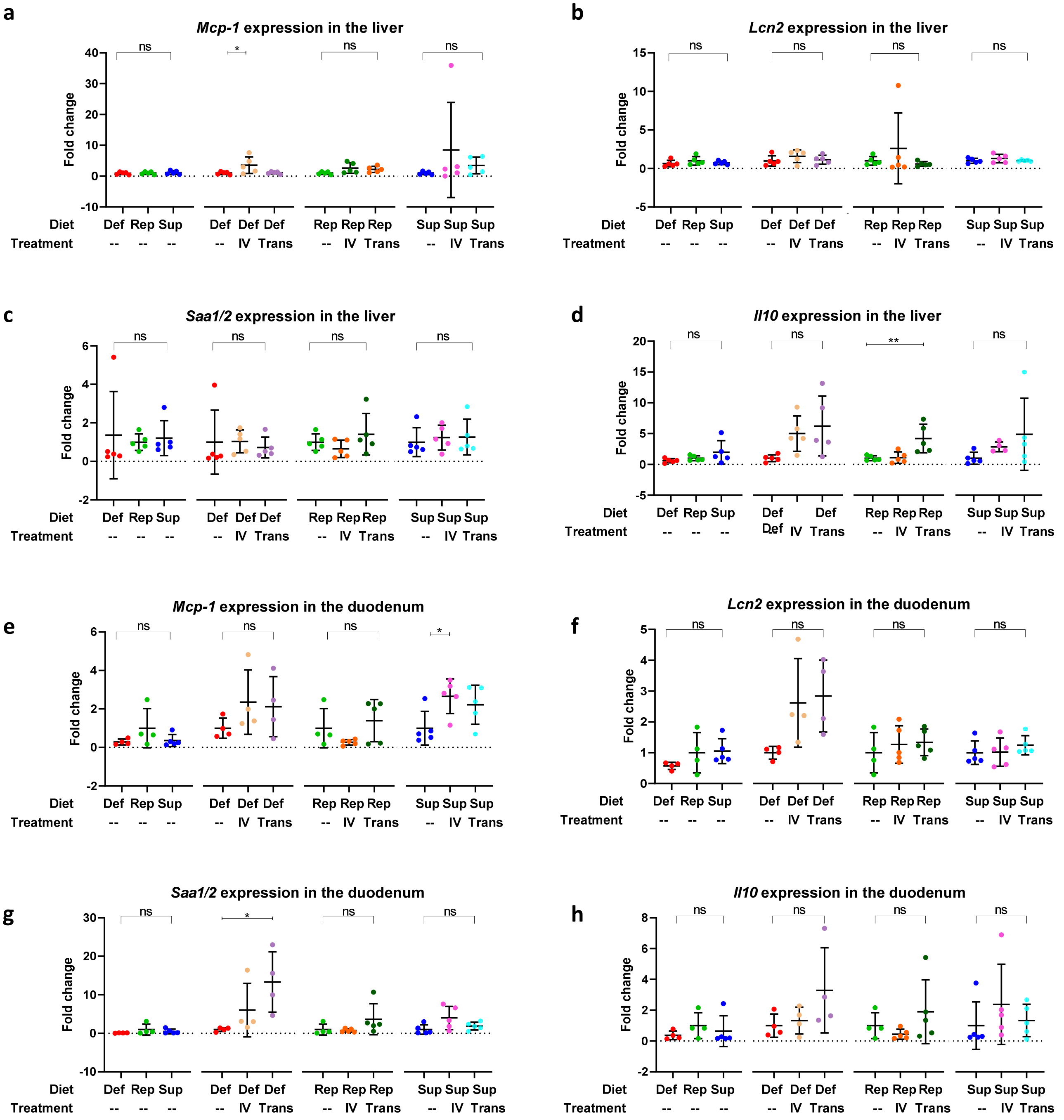

Supplementary Figure 2. qPCR of pro and anti-inflammatory molecules in liver and duodenum. Dot plot graphs representing the fold change of monocyte chemoattractant protein (*Mcp)-1*, lipocalin (*Lcn)2*, serum amyloid A (*Saa)1/2* and interleukin (*Il)-10* molecules in **a-d** liver and **e-h** duodenum of one representative experiment (n=4-5 mice per group). For the fold change

calculation, the group of mice fed an iron-replete diet was used as control for the comparison of cohorts of mice fed diets alone. Cohorts of mice fed the respective diets alone were used as the controls for the comparison with mice chronically transfused or infused with intravenous (IV) iron. Values indicate mean ± SD. *p<0.05, **p<0.01, ***p<0.001, ****p<0.0001 by one-way ANOVA with Tukey’s multiple comparison test.

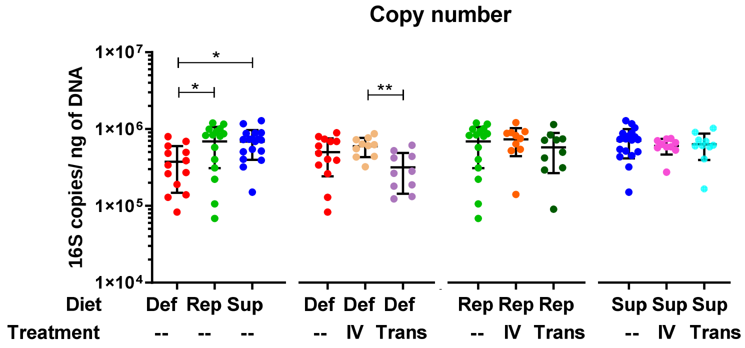

Supplementary Figure 3. 16S copy number analysis. 16S copy number analysis was performed by quantitative PCR on DNA extracted from cecal feces. Scatter plots show mean ± SD of values calculated combining data from 4 complementary experiments (n=10-20 per group). *p<0.05, **p<0.01 by one-way ANOVA with Tukey's multiple comparison test

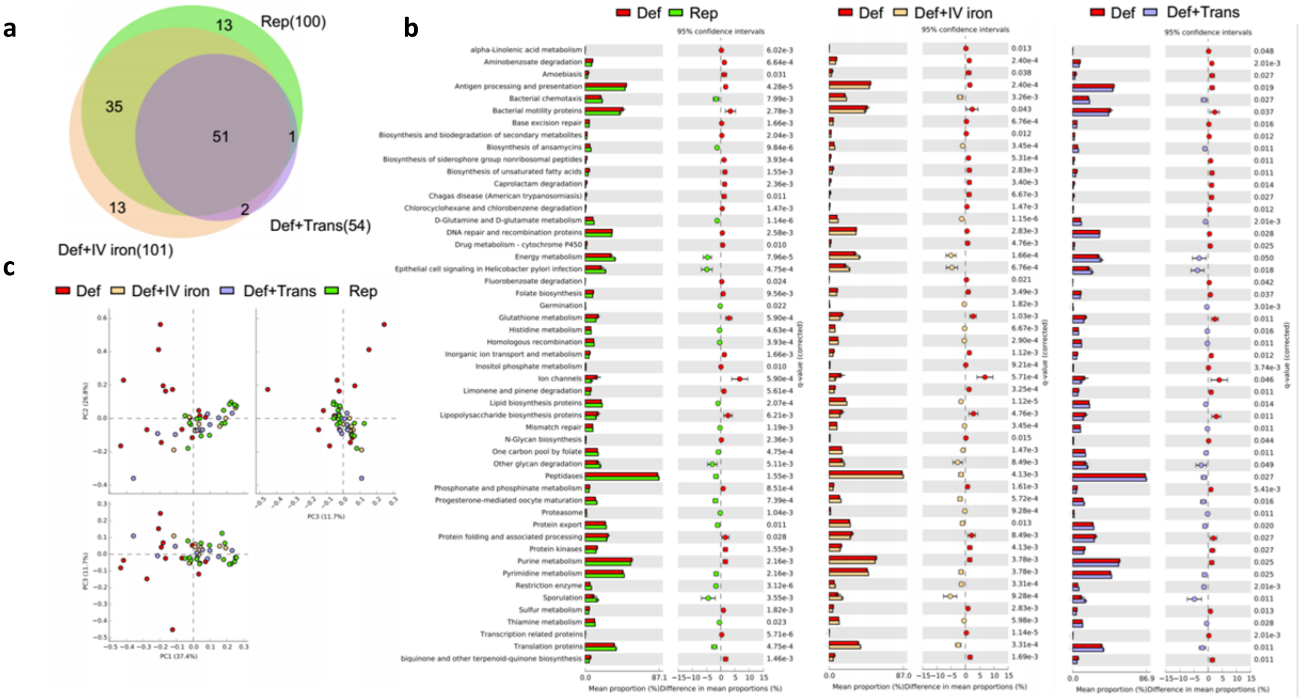

Supplementary Figure 4. Functional pathways predicted with PICRUSt analysis. **a** Venn diagram of the distribution of the predicted KEGG pathways at level 3 in cohorts of mice fed an iron-replete diet (Rep), or fed an iron-deficient diet and infused with intravenous iron (Def+IV iron) or chronically transfused (Def+Trans), as compared to the cohort of mice fed an iron-deficient diet alone (Def); **b** Mean proportion and differences of the 51 common pathways found to be significantly enriched or depleted in the 3 comparisons at KEGG level 3. The q-values are calculated using Welch's t-test with Storey's false discovery rate comparison. **c** Principal component analysis (PCA) of predicted functional pathways at KEGG level 3. n=10-20 mice per group.

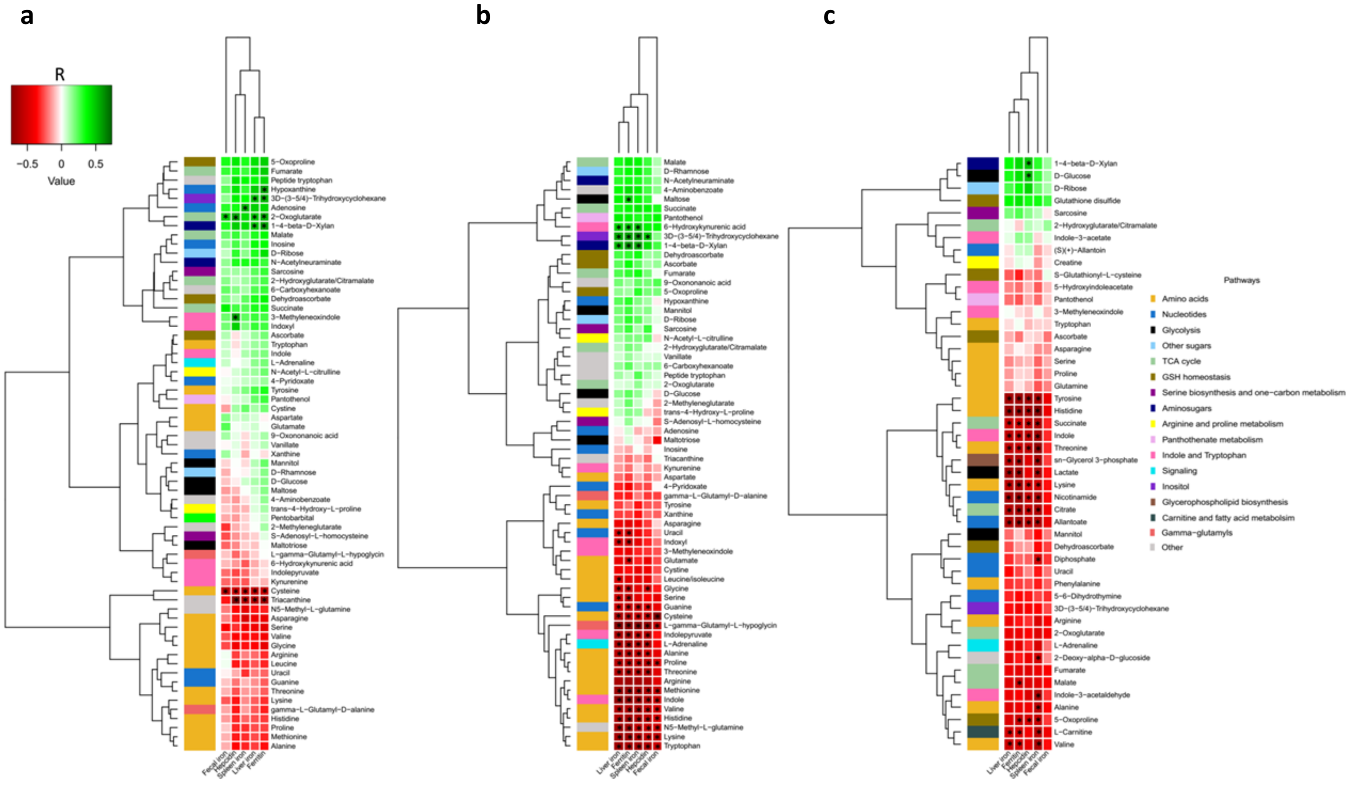

Supplementary Figure 5. Correlation of metabolites with iron status. Heatmap of Spearman's correlation between iron parameters and detected metabolites in two separate experiments (n=60 mice total) in **a** cecal feces, **b** stool pellets, and **c** plasma. *p<0.0001 for Spearman's correlation.

Supplementary Table 1. Mixed model analysis of the Shannon alpha diversity index

Mixed model random effect "Experiment"			Parameter	Diet	Treatment	Estimate	SE	DF	t	p-value
Fixed effects	F Value	Pr>F	Intercept			6.1637	0.4679	3	13.2	**0.0009**
Diet	24.13	**<.0001**	Diet	Def		-0.5687	0.0961	102	-5.9	**<.0001**
			Diet	Sup		0.05721	0.1079	102	1.4	0.597
			Diet	Rep		0	.	.	.	.
Treatment	18.77	**<.0001**	Treatment		IV iron	0.5918	0.101	98	2.83	**<.0001**
			Treatment		Trans	0.4174	0.101	98	3.03	**<.0001**
			Treatment		none	0	.	.	.	.

Mixed model analysis results considering diet and treatments (intravenous iron infusion and chronic transfusion) as fixed effects and each single experiment as a random effect and multiple comparison results comparing each treatment and diet to the cohorts of mice fed an iron-replete diet.
